# Supplementary material for: Causal Relationship between Aging and Anorexia Nervosa: A White-Matter-Microstructure-Mediated Mendelian Randomization Analysis
Source: Biomedicines. 2024 Aug 16;12(8):1874. doi: 10.3390/biomedicines12081874 (PMC11351342; doi:10.3390/biomedicines12081874)
Supplement: Supplementary file 1 [file biomedicines-12-01874-s001.zip › Supplementary Figure/Figure S4/Singe SNP plot of MR analysis for TL to ubm-b-1602.pdf]

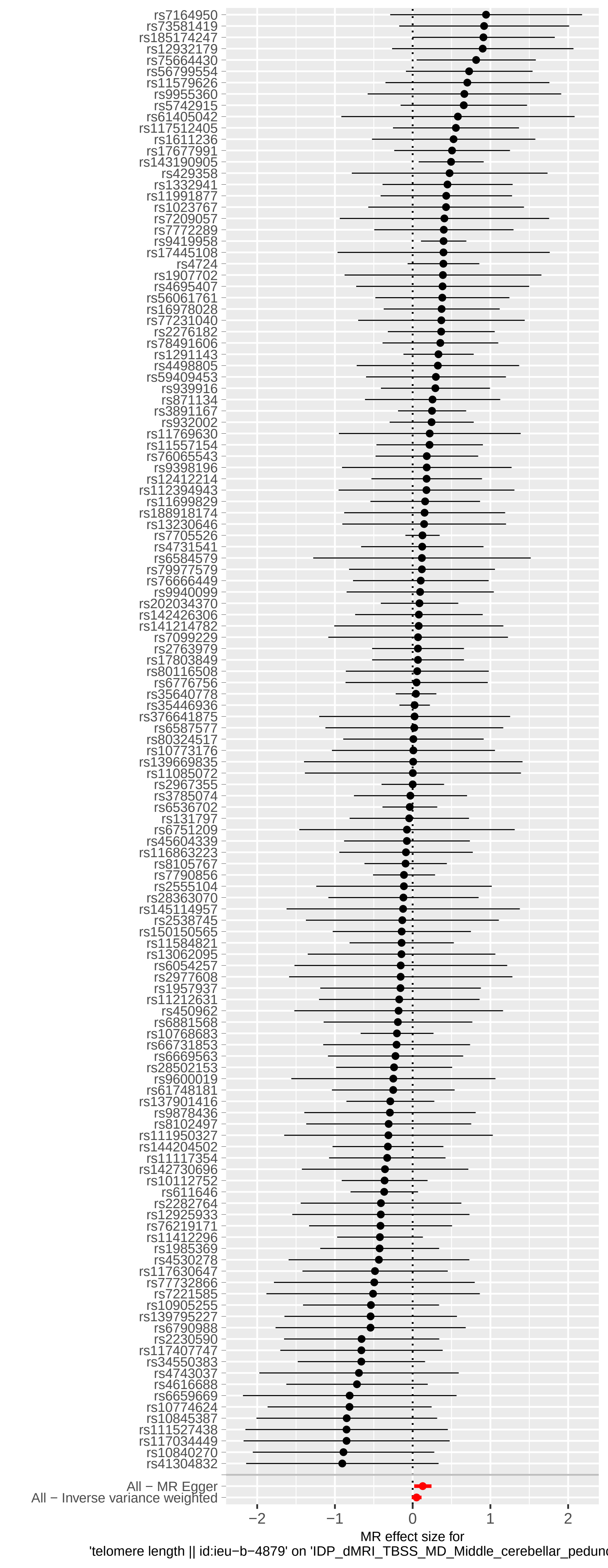
rs7164950
rs73581419
rs185174247
rs12932179
rs75664430
rs56799554
rs11579626
rs9955360
rs5742915
rs61405042
rs117512405
rs1611236
rs17677991
rs143190905
rs429358
rs1332941
rs11991877
rs1023767
rs7209057
rs7772289
rs9419958
rs17445108
rs4724
rs1907702
rs4695407
rs56061761
rs16978028
rs77231040
rs2276182
rs78491606
rs1291143
rs4498805
rs59409453
rs939916
rs871134
rs3891167
rs932002
rs11769630
rs11557154
rs76065543
rs9398196
rs12412214
rs112394943
rs11699829
rs188918174
rs13230646
rs7705526
rs4731541
rs6584579
rs79977579
rs76666449
rs9940099
rs202034370
rs142426306
rs141214782
rs7099229
rs2763979
rs17803849
rs80116508
rs6776756
rs35640778
rs35446936
rs376641875
rs6587577
rs80324517
rs10773176
rs139669835
rs11085072
rs2967355
rs3785074
rs6536702
rs131797
rs6751209
rs45604339
rs116863223
rs8105767
rs7790856
rs2555104
rs28363070
rs145114957
rs2538745
rs150150565
rs11584821
rs13062095
rs6054257
rs2977608
rs1957937
rs11212631
rs450962
rs6881568
rs10768683
rs66731853
rs6669563
rs28502153
rs9600019
rs61748181
rs137901416
rs9878436
rs8102497
rs111950327
rs144204502
rs11117354
rs142730696
rs10112752
rs611646
rs2282764
rs12925933
rs76219171
rs11412296
rs1985369
rs4530278
rs117630647
rs77732866
rs7221585
rs10905255
rs139795227
rs6790988
rs2230590
rs117407747
rs34550383
rs4743037
rs4616688
rs6659669
rs10774624
rs10845387
rs111527438
rs117034449
rs10840270
rs41304832
All − MR Egger
All − Inverse variance weighted
−2
−1
0
1
2
MR effect size for
'telomere length || id:ieu−b−4879' on 'IDP_dMRI_TBSS_MD_Middle_cerebellar_pedunc
